# Bibliometric evaluation of *Anais Brasileiros de Dermatologia* (2013-2022)^⋆^

**DOI:** 10.1016/j.abd.2023.08.003

**Published:** 2023-09-27

**Authors:** Hélio Amante Miot, Paulo Ricardo Criado, Caio César Silva de Castro, Mayra Ianhez, Carolina Talhari, Paulo Müller Ramos

**Affiliations:** a Department of Infectology, Dermatology, Diagnostic Imaging and Radiotherapy, Universidade Estadual Paulista, Botucatu, SP, Brazil; b Centro Universitário Faculdade de Medicina do ABC, Santo André, SP; and Faculdade de Ciências Médicas de Santos (Fundação Lusíada), Santos, SP, Brazil; c Hospital de Dermatologia Sanitária do Paraná and Escola de Medicina, Pontifícia Universidade Católica do Paraná, Curitiba, PR, Brazil; d Department of Dermatology, Hospital de Doenças Tropicais de Goiás, Goiânia, GO, Brazil; e Department of Dermatology, Universidade do Estado do Amazonas, Manaus, AM, Brazil

**Keywords:** Bibliometrics, Impact factor, Journal article, Journal impact factor

## Abstract

The Anais Brasileiros de Dermatologia, published since 1925, is the most influential dermatological journal in Latin America, indexed in the main international bibliographic databases, and occupies the 50th position among the 70 dermatological journals indexed in the Journal of Citations Reports, in 2022. In this article, the authors present a critical analysis of its trajectory in the last decade and compare its main bibliometric indices with Brazilian medical and international dermatological journals. The journal showed consistent growth in different bibliometric indices, which indicates a successful editorial policy and greater visibility in the international scientific community, attracting foreign authors. The increases in citations received (4.1×) and in the Article Influence Score (2.9×) were more prominent than those of the main Brazilian medical and international dermatological journals. The success of *Anais Brasileiros de Dermatologia* in the international scientific scenario depends on an assertive editorial policy, on promptly publication of high-quality articles, and on institutional stimulus to encourage clinical research in dermatology.

## Introduction

Scientific knowledge production in the biomedical area must directly reach society, health professionals, and researchers through the publication of peer-reviewed scientific research, so it can be consulted, criticized, operationalized, improved and submitted for refutation.^1^, ^2^ Thus, scientific articles make up the main cell of biomedical science, and scientific journals constitute the great vectors of modern science. The quality of a scientific article is a multidimensional concept, which encompasses its degree of originality, the objectivity and clarity of the text, methodological and analytical property, interest (social or academic), transparency, and potential replicability, in addition to contextualization with available knowledge.^3^, ^4^ Then, it is expected that the highest quality journals are those that aggregate the best scientific articles.^5^

However, scientific journals reflect particularities regarding the area of knowledge, the characteristics of the research they publish, the type of scientific article, and even the research capabilities in the host country. These are important points in journal ranking, especially when comparing papers from different medical specialties published in non-English languages, and reflect the reality of research in different countries, such as Brazil.^6^, ^7^

Brazilian scientific research is still based on postgraduate programs in public universities, largely dependent on government funding agencies, with little participation from private research institutions.^8^, ^9^ These points themselves justify the lower volume and lower international impact of Brazilian scientific research as a whole.^6^, ^10^, ^11^

The evaluation of the quality of scientific journals also depends on different qualitative and quantitative elements, and different bibliometric indices help in the perception of their scientific influence, subsidizing decisions regarding editorial policies.^12^, ^13^

This article discloses a critical analysis of the trajectory of the Anais Brasileiros de Dermatologia (ABD) in the last decade; the authors compare its main bibliometric indices with Brazilian medical and international dermatological journals.

## Fundamental analysis

ABD is a well-established scientific journal, published uninterruptedly since 1925 by the Brazilian Society of Dermatology (SBD, *Sociedade Brasileira de Dermatologia*), an organization representing Brazilian specialists. It has editorial independence, and the Chief Editor is elected in secret voting by a collegiate of representatives of SBD members, every five years. Moreover, it has a qualified editorial board consisting of 60 members from 14 countries/regions, 32 of which are from Brazil (https://www.anaisdedermatologia.org.br/pt-comite-editorial).

The journal is indexed in the main bibliographic databases: PubMed/PMC/MEDLINE, Scopus, ISI-Web of Science, SciELO, Lilacs, Embase (Excerpta Médica), and Latindex, established as the most influential Latin American dermatological journal, and the only one indexed in MEDLINE database.^14^

All articles are peer-reviewed, anonymously (as for authors and institutions), using an electronic system, after free submission. The accepted, corrected and formatted articles are made available online (ahead of print) until inclusion in an edition. Issues are published bimonthly, in Portuguese (printed and online on the journal website) and in English (online), with open public access to the entire collection.

The ABD constitutes a source of knowledge dissemination in dermatology and related specialties, emphasizing tropical dermatology, infectious and parasitic dermatology, sexually transmitted infections, AIDS, and dermatopathology. For this purpose, there are sections for review papers, continuing medical education articles, original (investigative) articles, research letters, clinical case letters, dermatopathology letters, tropical/infectious-parasitic dermatology letters, and special articles. They also offer space for letters to the editor, allowing the publication of critical of the published articles.

In recent years, ABD has shown a consistent increase in different bibliometric indices in the national and international scenario,^12^ leading ABD to be classified as QUALIS B2, in the current classification (2020) of the Coordination for the Improvement of Higher Education Personnel (CAPES, *Coordenação de Aperfeiçoamento de Pessoal de Nível Superior*), the normative body for postgraduate programs in Brazil.

According to the Impact Factor (IF) of 2022, which considers two years of publications, the ABD occupies the 50th position among 70 dermatological journals indexed by the JCR ‒ Journal of Citations Reports (https://jcr.clarivate.com/jcr-jp/journal-profile?journal=AN%20BRAS%20DERMATOL&year=2022), ranking in the third classification quartile of the category. However, according to the SCOPUS database cite score (https://www.scopus.com/sourceid/24247), which considers four years of publications, it ranks 60th among 133 indexed dermatological journals, placing it in the second quartile. When compared to the 32 Brazilian medical journals indexed in the JCR, the ABD occupies the 18th position.

In the analyzed decade, according to the IF hierarchy, the ABD advanced from 20% to 29% in the ranking of international dermatological journals, and from 26% to 44% among Brazilian medical journals (Table 1). This increase was more noticeable from 2020 onwards. Despite the great interest in cosmetic related reports in the last two decades, the ABD continued to encompass broad aspects of the specialty, aiming the training and recycling of dermatogists or medical colleagues interested in different areas of clinical, sanitary, surgical, and cosmetic subjects, in addition to basic science.

**Table 1:** Main bibliometric indicators of *Anais Brasileiros de Dermatologia* (2013‒2022)

Year	Immediacy index	IF (2y)	IF (5y)	Eigenfactor Score	Normalized Eigenfactor	Influence score	DERM^a^	MED-BR^a^	P/C – DERM^b^	Full articles^c^	Received citations	Self-citation (2y)	Half-life of citations	Age of references
2022	0.775	1.724	1.974	0.00286	0.61560	0.465	29%/70	44%/32	8/374	65	3.550	4%	6.7	7,3
2021	0.412	2.113	2.290	0.00352	0.75772	0.465	34%/69	52%/33	9/393	86	3.855	5%	6.1	7,8
2020	0.372	1.896	2.057	0.00408	0.85607	0.445	27%/69	62%/34	11/377	105	3.520	6%	5.7	7,5
2019	0.204	1.121	1.514	0.00395	0.48152	0.333	17%/68	29%/24	7/283	101	2.585	6%	5.6	8,2
2018	0.125	1.050	1.398	0.00424	0.50537	0.320	16%/66	26%/23	7/261	140	2.333	6%	5.2	8,3
2017	0.064	0.884	1.242	0.00342	0.39916	0.273	7%/64	22%/23	7/245	201	1.847	7%	4.8	8,5
2016	0.048	0.978	1.091	0.00353	0.40585	0.260	15%/63	33%/24	7/228	129	1.456	8%	4.7	7,9
2015	0.074	0.880	1.053	0.00344	0.39195	0.261	14%/61	27%/22	7/197	190	1.282	12%	4.4	8,5
2014	0.130	0.723	0.918	0.00237	0.26552	0.185	15%/63	17%/23	7/195	135	1.077	24%	4.1	8,2
2013	0.048	0.866	0.804	0.00175	0.19259	0.161	20%/61	26%/23	7/189	207	867	28%	3.8	8,1

a Percentile occupied by ABD among journals in the category: DERM (International Dermatology), MED-BR (Brazilian Medicine).

b P/C – DERM: Published Full Articles/Category Citations (×1000).

c Full-text articles: Reviews + Original articles (excluding editorials and letters).

In 2022, it received 641 submissions, of which only 171 (27%) were accepted for publication. The mean time between article submission and acceptance was 14 weeks; however, the interval between submission and online publication was 64 weeks, which is long in terms of scientific dissemination.

From the structural point of view, in 2022 it published 15 reviews, 65 original articles, and 86 other papers (e.g., letters and editorials), which are citable but not considered full articles. Among the 20 most cited articles in 2022, 10 (50%) of them were review or consensus articles, with no clear differences among the most cited topics (acne, oncology, atopic dermatitis, surgery, phototherapy, psoriasis, vitiligo, melasma, and leprosy).^15^, ^16^, ^17^, ^18^, ^19^, ^20^, ^21^, ^22^, ^23^, ^24^, ^25^, ^26^, ^27^, ^28^, ^29^, ^30^, ^31^, ^32^, ^33^, ^34^ The references listed in ABD articles had a median age of 7.3 years. Furthermore, half of the citations of ABD articles, in 2022, were more recent than 6.7 years.

**Figure 1:**
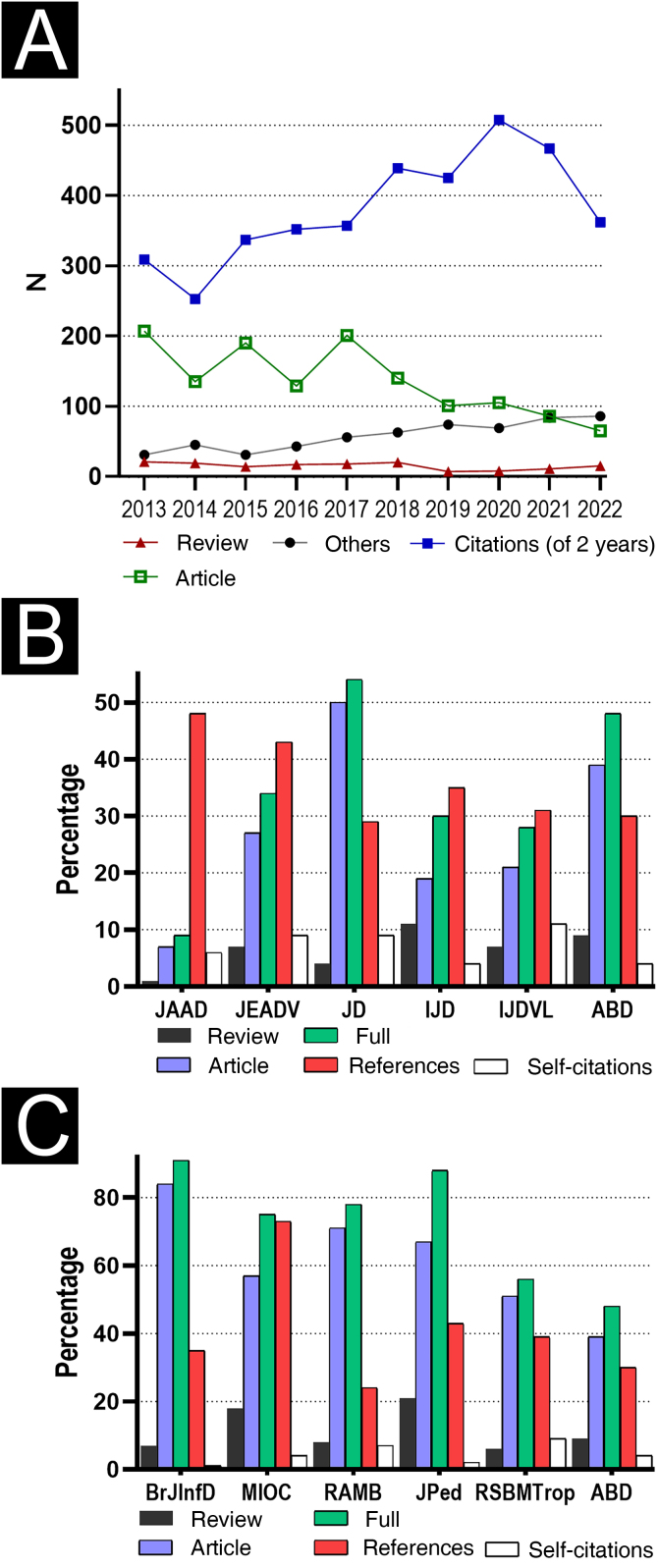
(A) Time series of citations of articles published in the last two years, review articles, full articles, and other publication items, in the 2013-2022 decade for ABD (*Anais Brasileiros de Dermatologia*). (B) Ratios of review articles, original articles, full articles among all publications, references to full articles, and self-citations in relation to all citations from 2022 international dermatological journals: Journal of the American Academy of Dermatology (JAAD), International Journal of Dermatology (IJD), Journal of Dermatology (JD), Journal of the European Academy of Dermatology and Venereology (JEADV) and Indian Journal of Dermatology Venereology and Leprology (IJDV). (C) Comparison between national clinical journals: *Memórias do Instituto Oswaldo Cruz* (MIOC), *Revista da Associação Medica Brasileira* (RAMB), *Jornal de Pediatria* (JPed), *Revista da Sociedade Brasileira de Medicina Tropical* (RSBMtrop), Brazilian Journal of Infectious Diseases (BJID)

Fig. 1 shows the percentage of full articles (original articles and reviews) among all published items (2022) since only full articles constitute the denominator for IF calculation. Moreover, it shows the proportion of references per full article for the ABD, and those related to Brazilian medical and international dermatological journals, in 2022. As references for international comparison, the Journal of the American Academy of Dermatology (JAAD) was chosen because it is the dermatological publication with the highest IF in the last decade; the International Journal of Dermatology (IJD) and the Indian Journal of Dermatology, Venereology and Leprology (IJDV) were selected for having editorial characteristics similar to the ABD; the Journal of Dermatology (JD) and the Journal of the European Academy of Dermatology and Venereology (JEADV) for being journals that represent the Japanese and one European society of dermatology. The references for Brazilian journals were clinical and tropical medicine publications that most closely resemble the editorial profile of the ABD: *Memórias do Instituto Oswaldo Cruz* (MIOC), *Revista da Associação Médica Brasileira* (RAMB), *Jornal de Pediatria* (JPed), *Revista da Sociedade Brasileira de Medicina Tropical* (RSBMtrop), and the Brazilian Journal of Infectious Diseases (BJID).

For ABD, both general citations (-8%) and articles published in the last two years (-22%) showed a decline in 2022. The proportion of reviews (15%) among published items is still below that of 2018. There was, however, a progressive reduction in the proportion of full articles to the detriment of other citable items (editorials and letters).^35^

When compared to other dermatological journals, ABD had lower proportions of self-citations (4% of citations), and a lower number of references per full article (mean of 30 references/article). However, ABD stood out for its high percentage of full articles in relation to the total number of items published in the period (48%), which has been gradually decreasing in the last decade. Considering the national scenario, the lowest number of references per full article and the lowest proportion of self-citations stand out.

Most of the articles published in 2022 were written by Brazilian authors (61%), especially from public universities, followed by authors from Turkey, Spain, China, and Portugal (17%). The citations received by ABD, in 2022, were mostly foreign, mainly from JEADV, Dermatology and Therapy, Cureus, IJD, Journal of Fungi, Frontiers in Medicine, and Clinical Cosmetic and Investigational Dermatology. Self-citations decreased by 86% in the analyzed decade, which indicates the strong internationalization of the journal impact.

## Bibliometric analysis of the Anais Brasileiros de Dermatologia

There is no absolute and objective way to measure the influence of a journal in its area of knowledge, or even in the scientific universe as a whole. While there are no reliable sources to assess individual access to texts, their dissemination, or their effects, the core of bibliometric indicators is based on citations that the journal receives from other publications.^36^, ^37^

In time, citations can be evaluated by considering the bibliographic bases that record them, the nature of the journal that cited them, the country of origin of the citation, the year of the published article, the number of articles published by the journal under analysis, the behavior of other citations of journals in the database, among other covariates. These different nuances generate the main bibliometric indices, which may have different interpretations, and require caution in their analysis.^36^

The IF is the most frequently used metric to assess the relevance of an academic journal. It is calculated each year from the sum of citations received in that year (e.g., 2022) from articles published in the previous two years (e.g., 2020 and 2021), divided by the number of articles published in the previous two years (e.g. 2020 and 2021).^36^, ^38^ However, there is also criticism of the exclusive use of the IF to assess research quality, as it may favor journals that publish certain types of articles that are more frequently cited, such as reviews and consensuses, to the detriment of other types of research that are equally valuable but may receive fewer citations, such as case reports.^39^, ^40^ Alternatively, the IF can be calculated for five-year citations.

The Immediacy Index measures how quickly articles published in a scientific journal are cited right after their publication. It is calculated by dividing the total number of citations received by articles published in a given year by the number of these articles published in that same year as if it were an instantaneous IF.^36^

The Eigenfactor score is a metric that not only takes into account the citations received over a five-year period, but also the importance of the journals that make these citations.^36^ The Eigenfactor does not consider self-citations; it is calculated based on an algorithm that takes into account the structure of the citation network among journals. It can be normalized (Normalized Eigenfactor) to take into account the size and nature of the journal, and this allows comparing journals of different sizes or areas of knowledge, providing a more comprehensive view of the relevance and reach of a journal in the scientific community.

As both the IF and the Eigenfactor are inflated by the number of publications of a journal, the article Influence Score uses the Normalized Eigenfactor adjusted by the journal publication volume.

The JCR database, although expanding annually, counted in 2022 with 21,522 scientific journals. The SCOPUS database, on the other hand, encompasses 44,737 journals (peer-reviewed ones), online books, and conference publications, explaining its greater scope. The analysis of the bibliometric data in Table 1 shows the main indices of ABD, in the last decade. The Immediacy Index indicator showed the most prominent growth (×16), followed by the total number of citations received (×4), normalized Eigenfactor (×3), and the influence score (×3). ABD the IF (2- and 5-year) and the Eigenfactor score showed a more modest growth in the decade and a reduction in the last year. Of particular note is the marked increase in publications and citations since 2020 in all medical categories, which may have been prompted by the COVID-19 pandemic.^41^

**Figure 2:**
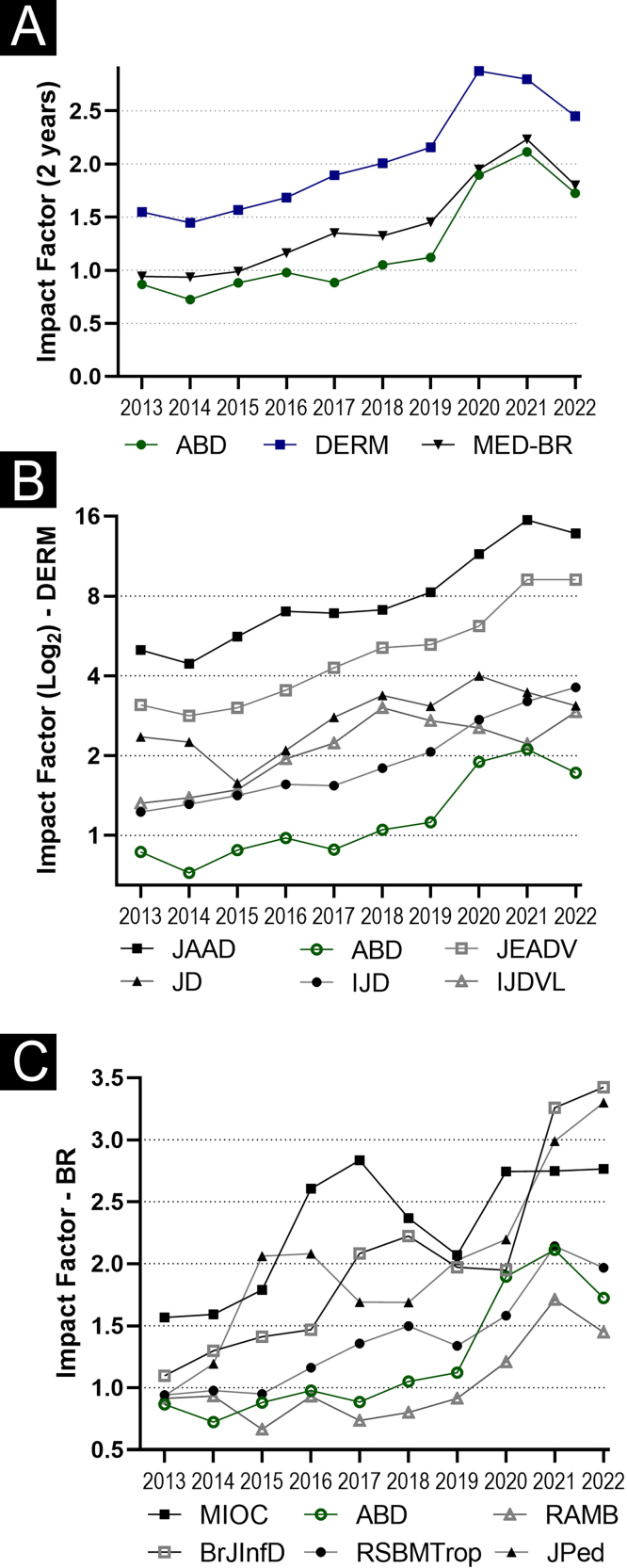
Time series of Impact Factors (IF) in the 2013‒2022 decade. (A) *Anais Brasileiros de Dermatologia* (ABD), indexed International Dermatological Journals (DERM), and indexed Brazilian Medical Journals (MED-BR). (B) Series with the IF of International Dermatological Journals: Journal of the American Academy of Dermatology (JAAD), International Journal of Dermatology (IJD), Journal of Dermatology (JD), Journal of the European Academy of Dermatology and Venereology (JEADV) e Indian Journal of Dermatology Venereology and Leprology (IJDV). (C) Series with the IF of national clinical journals: *Memórias do Instituto Oswaldo Cruz* (MIOC), *Revista da Associação Médica Brasileira* (RAMB), *Jornal de Pediatria* (JPed), *Revista da Sociedade Brasileira de Medicina Tropical* (RSBMtrop), Brazilian Journal of Infectious Diseases (BJID)

Table 1 details the evolution of ABD different bibliometric indices in the last decade, and Fig. 2 shows the evolution of the IF of ABD, compared to indexed Brazilian medical and international dermatological journals. When comparing the progression of the IF of ABD with the median of the international dermatological journals, and of the Brazilian medical journals indexed in the JCR, there is evidence of consistent growth of the IF in the global scenario of the last decade: ABD (99%), international dermatological journals (58%), and Brazilian medical journals (91%). The linear growth remains, despite the reduction in the 2022 IF: ABD (-18%), international dermatological journals (-12%), and Brazilian medical journals (-19.3%).

**Figure 3:**
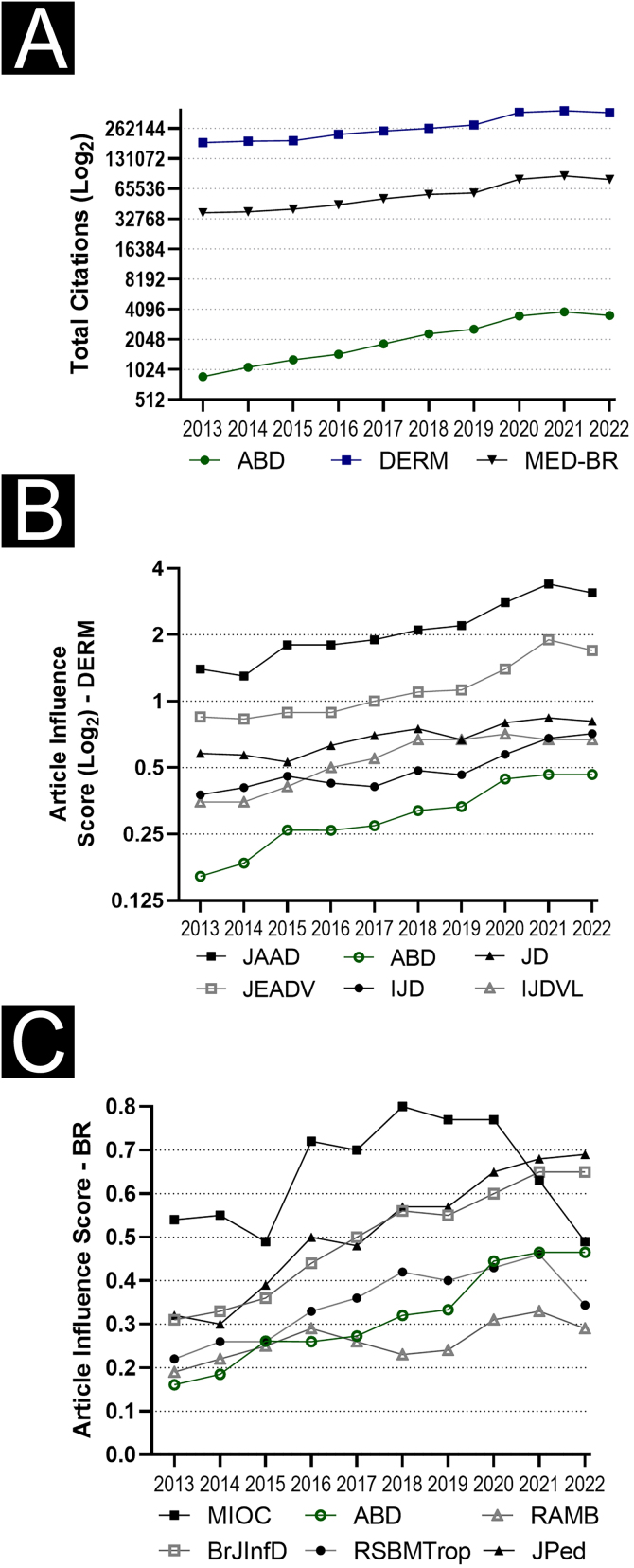
(A) Time series of total citations received per year in the 2013‒2022 decade for *Anais Brasileiros de Dermatologia* (ABD), Indexed International Dermatological Journals (DERM), and Indexed Brazilian Medical Journals (MED-BR). (B) Series with article influence scores of international dermatological journals: Journal of the American Academy of Dermatology (JAAD), International Journal of Dermatology (IJD), Journal of Dermatology (JD), Journal of the European Academy of Dermatology and Venereology (JEADV) and Indian Journal of Dermatology Venereology and Leprology (IJDV). (C) Series with the article influence scores of national clinical journals: *Memórias do Instituto Oswaldo Cruz* (MIOC), *Revista da Associação Médica Brasileira* (RAMB), *Jornal de Pediatria* (JPed), *Revista da Sociedade Brasileira de Medicina Tropical* (RSBMtrop), Brazilian Journal of Infectious Diseases (BJID)

Fig. 3 shows the citations received by ABD, Brazilian medical journals, and international dermatological journals in 2022. In addition, it compares the influence scores of articles published by some Brazilian medical and international dermatological journals. ABD showed, in the studied decade, an increase in the number of citations (×4.1), which was higher than that of indexed dermatological journals (×2.0) and indexed Brazilian medical journals (×2.1). Similarly, the growth (×2.9) of ABD influence score was greater than that of selected Brazilian medical journals and selected international dermatology journals.

## Discussion

The volume of scientific journals grows annually, which requires the use of bibliometric indicators for an adequate ranking of their relevance. In the last decade, the massive growth in the number of citations of ABD, the immediacy with which the publications were cited, and the greater influence of the articles outperformed international dermatological and Brazilian medical journals. These elements reflect success in editorial policy and indicate consistency in the increase of its influence.

The IF and Eigenfactor scores are greatly influenced by the total number of items published in each assessment year and showed a reduction in 2022 for dermatological journals as a whole. There was, however, a 22% reduction in articles published in all dermatological journals after the COVID-19 pandemic (10,610/2020, to 8,248/2022) penalizing them regarding these items. In these terms, ABD can benefit from the increase in the number of articles published per issue, since they already accumulate a notable rejection rate (73%); however, their volume of publications is much lower than the journals used for the comparison of this article.

Likewise, encouraging the use of more recent references, and in greater numbers per article, is an element that can benefit its visibility. It was observed that the number of bibliographic references in an article, the up-to-dateness of the references (< 5 years), and the length of the abstract were associated with a greater chance of citation.^42^, ^43^ It should also be noted that the number of self-citations of the articles in ABD is very low, which may represent the amplitude of topics it addresses, and the lack of well-defined research lines of South American dermatologists.

The ABD should benefit from the speed of online availability (ahead of print) of the main accepted articles. Prestigious dermatological journals such as JAAD and JEADV have the accepted texts, without formatting, a few days after acceptance. The delay of almost one year for the online publication of accepted articles reduces the visibility and originality of the research, misses the opportunity to be cited, makes the bibliographical research out of date, in addition to not participating in the discussion of emerging issues (e.g., epidemics of COVID-19 and monkeypox). Increasing the number of articles in each issue can also reduce the waiting list for publication.

The compulsory adoption of publication recommendations, such as CARE (case reports), STROBE (observational studies), CONSORT (clinical trials), and PRISMA (systematic reviews) should increase the quality of scientific communications of ABD; moreover, the adoption of checklists of these guidelines is recommended for both authors and reviewers.^44^

ABD can benefit from publishing letters and editorials to deal with occasional communications, rather than full articles. There is a recent movement by a number of journals to prioritize the use of letters in the dissemination of less robust studies, or with a smaller number of variables involved, which demand less need for discussion.^45^ In 2022, the JAAD published only 9% of the 1,777 items as full articles; the JEADV, 34% of 883 items; the IJD, 30% of 515 items, while the ABD published 48% of 166 items. This editorial strategy does not penalize the journal’s IF, since letters are not considered articles that add up to the calculation denominator; on the contrary, these publications potentially amplify their citations, and may inflate their value.^35^ Furthermore, neither the length of the article nor the number of authors is associated with the number of citations it produces.^46^

ABD can encourage the invitation of prolific researchers with a well-structured line of research to write good review articles on their fields of study. Review articles have high educational visibility and return citations – especially – by well-defined research groups.^47^, ^48^ In the meantime, expert consensuses are also valuable regarding their up-to-dateness and applicability. The abundance of clinical, surgical and sanitary conditions with which dermatology deals creates a favorable environment for the publication of reviews and consensuses. Involving productive authors in their review and consensus articles maximizes the citation of their own productivity in future publications.

On the other hand, Brazilian researchers linked to public research institutions are evaluated according to their productivity in publications with higher IF. This is a factor that naturally brings disadvantages for national journals, as they have lower IF, leaving them with lower quality articles, which generate fewer citations and – in a vicious circle, not at all a virtuous one – do not boost national journals.^49^

As ABD publications are open access, investments should be made in increasing the visibility of their articles in the society, among dermatologists and research groups. Faced with the myriad of scientific publications available, the journal's greater visibility must be sought by the adequate indexing of its articles and metadata (e.g., keywords, DOI of references), which facilitates search by search systems.

Medical journals are also accessed by patients, disease support groups, health insurance companies, and journalists, among others who are interested in the published subject, and this social interest in published texts is not captured by bibliometric indicators. Altmetrics is an index that evaluates the penetration of a given publication in social networks such as Facebook, Twitter, Instagram, and sites such as the BBC and the Times. The dissemination of publications outside the medical sphere can be an option to ensure greater visibility of research, as well as a greater number of citations.^50^ Also, social media could be used to publicize the main articles of ABD, for instance, using the author’s own videos or graphic summaries.^51^

Finally, it is essential that the improvement of bibliometric indices of ABD should be based on the publication of articles of great scientific relevance. However, as a journal based in Brazil, it reflects the nature of Brazilian and Latin American medical research, which is still not very competitive internationally.^6^ Moreover, Brazilian dermatologists are geared towards individual clinical activity, are not very adept at postgraduate courses (Master and Doctorate degrees), and still have difficulty reading English.^52^ It is up to the SBD, together with its services, to stimulate scientific production during the medical residency, as an optional tool for the generation of original scientific knowledge in the specialty. After all, the country still experiences endemic dermatological conditions, in addition to lacking independent clinical research for the critical evaluation of the incorporation of new technologies and drugs into the health system.^53^, ^54^, ^55^

Although the number of researchers, research institutions, journals, and publications have increased in recent years, in Latin America, in general, science is not a fundamental part of its economy. The difference in regard to the scientific production of developed countries is still huge. Among the reasons for the low productivity of Latin American countries are limited financial resources for research, inadequate public budgets, below-standard levels of infrastructure and laboratory equipment, high cost of supplies/consumables, and inadequate remuneration of researchers and other human resources involved in research. Political as well as economic instability in most of these countries is a determining factor in the lack of long-term plans for scientific development in this region.^9^, ^52^ The initiatives of research funds for dermatological research (FUNADERM and FUNADERSP) of the SBD and SBD-RESP have already facilitated more than 70 research projects, and they are embryos that aim to promote the scientific development of the specialty, which must be valued.

In conclusion, the consistency of the improvement in the influence of ABD on the international scenario depends on a series of editorial actions that involve an effort related to the rigorous selection of articles and agile dissemination of approved texts. The alignment of this editorial policy must be carried out with the reviewers and communicated to the authors, aiming to maximize the quality of the published articles.

## Financial support

None declared.

## Authors’ contributions

Hélio Amante Miot: Design and planning of the study, drafting and editing of the manuscript; approval of the final version of the manuscript.

Paulo Ricardo Cria: Design and planning of the study, drafting and editing of the manuscript; approval of the final version of the manuscript.

Caio César Silva de Castro: Design and planning of the study, drafting and editing of the manuscript; approval of the final version of the manuscript.

Mayra Ianhez: Design and planning of the study, drafting and editing of the manuscript; approval of the final version of the manuscript.

Carolina Talhari: Design and planning of the study, drafting and editing of the manuscript; approval of the final version of the manuscript.

Paulo Müller Ramos: Design and planning of the study, drafting and editing of the manuscript; approval of the final version of the manuscript.

## Conflicts of interest

Hélio Amante Miot: Advisory Board – Johnson & Johnson, L’Oréal, Theraskin, Sanofi and Pfizer; Clinical research - Abbvie, Galderma and Merz.

Paulo Ricardo Created: Advisory board - Pfizer, Galderma, Takeda, Hypera, Novartis, Sanofi; Clinical research - Pfizer, Novartis, Sanofi, Amgen and Lilly; Lecturer: Pfizer, Abbvie, Sanofi-Genzyme, Hypera, Takeda, Novartis.

Caio César Silva de Castro: Advisory Board - Sanofi, Aché, Sun-Pharma, Galderma; Clinical Research - Abbvie, Pfizer, Jansen, Sanofi; Lecturer - Abbvie, Jansen, Novartis, Sanofi, Leo- Pharma.

Mayra Ianhez: Advisory Board - Galderma, Sanofi, Pfizer, Novartis, Abbvie, Janssen, UCB Biopharma, Boehringer-Ingelheim; Lecturer - Galderma, Sanofi, Pfizer, Theraskin, Novartis, Abbvie, Janssen, Leopharma, FQM.

Carolina Talhari: No conflicts of interest.

Paulo Müller Ramos: Advisory Board and lecturer – Pfizer.
